# Role of rotifer (*Brachionus plicatilis*) and *Artemia* (*Artemia salina*) nauplii in the horizontal transmission of a natural nervous necrosis virus (NNV) reassortant strain to Senegalese sole (*Solea senegalensis*) larvae

**DOI:** 10.1080/01652176.2020.1810357

**Published:** 2020-09-03

**Authors:** L. Vázquez-Salgado, J. G. Olveira, C. P. Dopazo, I. Bandín

**Affiliations:** Instituto de Acuicultura, Departamento de Microbiología y Parasitología, Universidade de Santiago de Compostela, Santiago de Compostela, Spain

**Keywords:** Antiviral activity, *Artemia salina*, *Brachionus plicatilis*, horizontal transmission, mortality, nervous necrosis virus, rotifer, Senegalese sole larvae, *Solea senegalensis*

## Abstract

**Background:**

Marine invertebrates are provided as a first feed for marine fish larvae because of their strict nutritional requirements, despite also being a potential source of infectious agents.

**Aim:**

To assess horizontal transmission of a nervous necrosis virus reassortant strain (NNV) to sole larvae via *Artemia* and rotifers.

**Materials and methods:**

Rotifer (*Brachionus plicatilis*) and *Artemia* (*Artemia salina*) nauplii cultures were bath infected with a reassortant (RGNNV/SJNNV) NNV strain isolated from gilthead sea bream and viral internalisation was confirmed by IFA. Senegalese sole (*Solea senegalensis*) larvae were fed on infected *Artemia* and disease signs and mortality were recorded. In addition, NNV viability was checked in cultures of either unfed invertebrates or invertebrates fed on phytoplankton and in the supernatant of microalgae cultures. All samples were tested by RT-qPCR and inoculation in cell culture.

**Results:**

Both rotifers and *Artemia* internalised NNV. Experimental transmission to sole larvae was achieved using infected *Artemia* and subsequently 60% mortality was recorded. At 24 h post-infection, orally infected individuals contained 9.34 × 10^4^ copies of viral RNA, whereas the bath infection yielded 2.05 × 10^6^ RNA copies larvae^−1^. Viral presence in both invertebrates was detected up to 8 days post infection but viral load decreased over time. Feeding with microalgae decreased viral detection even more and microalgae supernatants were demonstrated to significantly affect NNV viability.

**Conclusions:**

Our results demonstrate that both invertebrates can bioaccumulate NNV and that Senegalese sole larvae fed on infected *Artemia* might develop viral encephalopathy and retinopathy and high mortality.

## 1. Introduction

Disease outbreaks are the main threat to finfish and shellfish production in aquaculture hatcheries, resulting in major economic losses. It is therefore essential to increase our understanding of the aetiology of these outbreaks and to develop prophylactic measures to prevent the appearance of potentially pathogenic agents in these facilities.

Nowadays, cultured juvenile and adult fish are fed on commercial diets, mostly dry or semi-moistened pellets, which supply the nutrients and energy required to meet the physiological needs of growing animals in a safe manner (Hixson 2014). However, the strict food requirements of marine fish at early larval stages are not met by artificial feeding and, therefore, live food, consisting of marine invertebrates (zooplankton) such as rotifers or the brine shrimp, *Artemia salina*, enriched with microalgae, provide fish larvae with the essential nutrients for adequate development (Dhont et al. 2013; Eryalçın 2018, 2019). Although these invertebrates are a source of essential biomolecules, they can also act as pathogen vectors for healthy cultures, transmitting parasites, bacteria and viruses to the next link in the food web (Marcogliese 2002; Avila-Villa et al. 2011; Valverde et al. 2019), as demonstrated by the detection of the same bacterial population in fish guts and in live food (Turgay et al. 2020).

One of the most serious diseases affecting marine aquaculture is viral encephalopathy and retinopathy (VER) also known as viral nervous necrosis (VNN), caused by the nervous necrosis virus (NNV)—Genus *Betanodavirus*, Family *Nodaviridae*. NNV is a non-enveloped virus, composed of two molecules of single-stranded positive-sense RNA, namely RNA1 and RNA2 (Nagai and Nishizawa 1999). The current classification, based on a phylogenetic analysis of the T4 region in the RNA2 molecule, establishes four genotypes: barfin flounder nervous necrosis virus (BFNNV), red spotted grouper nervous necrosis virus (RGNNV), stripped jack nervous necrosis virus (SJNNV) and tiger puffer nervous necrosis virus (TPNNV) (Nishizawa et al. 1997). In addition, RGNNV and SJNNV reassortants in both forms (RGNNV/SJNNV and SJNNV/RGNNV, RNA1/RNA2) have been isolated in Southern Europe from farmed sole (*Solea* sp.), European sea bass (*Dicenthrarchus labrax*) and gilthead sea bream (*Sparus aurata*) (Bandín and Souto 2020). Betanodavirus transmission has been demonstrated to occur both horizontally and vertically (Doan et al. 2017; Bandín and Souto 2020). The horizontal route includes viral spreading through water during disease outbreaks, or by contact between infected fish or asymptomatic carriers and healthy susceptible animals. Several studies have suggested the possibility of non-fish carriers, reservoirs or vectors such as molluscs, crustaceans and rotifers, both in the wild and under farmed conditions (Skliris and Richards 1998; Gomez et al. 2006, 2008; Volpe et al. 2018).

The aim of this study was to assess whether a NNV reassortant strain (RGNNV/SJNNV) isolated from gilthead sea bream, could be transmitted through *Artemia* and rotifers to Senegalese sole larvae.

## 2. Materials and methods

### 2.1. Virus production and cell culture

A natural reassortant strain isolated from gilthead sea bream, with a RGNNV-type RNA1 and a SJNNV-RNA2, namely SpSaIAusc382.17 (hereafter 382.17), was propagated in E-11 cell line grown in L-15 medium containing 5% foetal bovine serum (FBS). Inoculated cells were maintained in L-15 medium supplemented with 2% FBS at 25 °C until the cytopathic effect (CPE) was complete. Then, the virus was clarified by centrifugation at 3000*g* for 15 min at 4 °C and stored at −80 °C until use. Viral titration was performed in 96-well plates incubated at 25 °C for 7–10 days. The plates were examined daily for the presence of CPE and the titres were expressed as 50% tissue culture infection dose per ml (TCID_50_ ml^−1^) (Reed and Muench 1938).

### 2.2. Rotifer and *Artemia salina* cultures and infection

Rotifer *Brachionus plicatilis* and brine shrimp *Artemia salina* cysts were kindly provided by Stolt Sea Farm S.A (Galicia, Spain). Rotifer stock were kept under continuous culture with sterile natural sea water (33 g l^−1^) and aeration, at a density of 80 rotifers ml^−1^. Artemia cysts were hatched in sterile natural sea water with strong aeration at a density of 60 cysts ml^−1^ and harvested at naupliar stage (the first developmental stage) after 24 h, separating nauplii from unhatched and empty cysts. In both cases, cultures were maintained in 1 l glass bottles at 22 °C in an illuminated room using a photoperiod of 16:8 light and dark cycle.

Both rotifers and *Artemia* nauplii were bath-challenged in triplicate with the 382.17 NNV strain at a concentration of 10^5^ TCID_50_ ml^−1^ for 24 h. After this period, they were rinsed in sea water to remove viral particles attached to the surface of the body and the remaining viral load was quantified. So as not to add more variables to the experiment, sub-cultivation was then performed with no food supply for 7 days. The length of the challenge was conditioned by zooplankton survival under the described conditions. Samples of 1 ml (80 rotifers ml^−1^ and 60 *Artemia* ml^−1^) were taken at 24 h, 3 and 7 days post rinsing (hpr and dpr, respectively), that is 48 h post infection (hpi), 4 and 8 days post infection (dpi). For comparative purposes, the invertebrates were fed once at 24 hpi with either *Nannochloropsis gaditana* or *Isochrysis galbana* cultures.

### 2.3. Sole infection challenges

Experimental infections in Senegalese sole (*Solea senegalensis*) larvae were performed in order to assess the invertebrates’ role in the transmission of this virus. Fish were handled in strict accordance with good animal practices regarding independently feeding larval forms as defined by EU guidelines (directive 2010/63/UE). The protocol was approved by the Galician Committee for experimental animal welfare and by the Xunta de Galicia (Permit Id. 15004/13/002).

A total of 550 Senegalese sole larvae (30-days post hatch -dph-) were used in this study. After an acclimatisation period at 22 °C, 10 larvae were killed with a tricaine methanesulfonate overdose (MS-222, Sigma-Aldrich, Darmstadt, Germany) and tested for the presence of nervous necrosis virus (NNV). The oral challenge was carried out on three groups of sole larvae (*N* = 80) by adding a dose of 60 NNV-infected *Artemia* nauplii per larvae, that were infected as described above, and subcultivated for 48 h before being fed to the larvae. Another three groups (*N* = 80) were bath challenged for 3 h at a viral concentration of 10^5^ TCID_50_ ml^−1^. In addition, two control tanks (*N* = 30) were set up, one for the bath infection and another for the oral infection. In the first, L-15 medium was used for mock infection and in the second fish were fed on non-infected *Artemia*. Seven larvae from each infected group were randomly sampled at 24, 72 hpi, 5 and 9 dpi. Larvae were observed twice a day and symptomatology and mortality were recorded. Both challenge experiments were terminated at 12 dpi. The sampled larvae, as well as the survivors, were euthanized as previously described. All the larvae were subjected to virological analysis and quantification of the viral load.

An oral challenge with an identical number of first feeding larvae (2 dph) and infected rotifers was also attempted. Unfortunately, sole larvae fed on them did not survive in our aquarium facilities enough time to complete the experiment.

### 2.4. Zooplankton immunofluorescence assay (IFA)

Infected zooplankton was cultivated as described above with no food supply for 72 h. Afterwards, they were fixed as follows: rotifers were incubated in paraformaldehyde 4% for 5 min, permeabilised as described above and blocked with skimmed milk (0.5% w/v) for 10 min (Kang et al. 2018). *Artemia* nauplii were fixed overnight in paraformaldehyde 4% (Panreac, Barcelona, Spain) at 4 °C, washed with 0.1% (v/v) Tween-80–phosphate-buffered saline (PBS), permeabilised with 0.1% (v/v) Triton X-100–PBS and blocked with skimmed milk (30% w/v for 30 min) (Cano et al. 2009). Finally, fixed invertebrates were incubated for 1 h with anti-NNV (ab26812–abcam) solution (ratio 1:10) and AntiRabbit IgG-FITC, and fluorescence was observed using an Eclipse T-2000 U inverted microscope (Nikon, Tokyo, Japan).

### 2.5. Microalgae culture and viral infection

*N. gaditana* (CCMP 526) and *I. galbana* (CCAP 927/20) were grown in natural, autoclaved and filtered sea water, and supplemented with GoldMedium (Aqualgae), following the manufacturer’s instructions. Cultures were maintained at 22 °C and 5400 lx light intensity, under a 16:8 light and dark photoperiod. Both microalgae cultures were started in test tubes (80 ml) and increased to 1 l glass bottles, at a density of 5 × 10^6^ cells ml^−1^ until reaching the stationary phase, which was used for zooplankton feeding. Microalgae culture growth was determined using a Neubauer counting chamber each day.

To assess the effect of microalgae water culture on viral viability, 50 ml of microalgae cultures at stationary phase were centrifuged at 4000*g* for 10 min, and the 382.17 strain was incubated in the supernatant at 25 °C. Triplicate samples were taken at 24, 72 h and 6 days of incubation and titrated as described above.

### 2.6. Sample processing and RNA extraction

Samples of whole *Artemia* and rotifers, and samples of larvae head (brain tissue and eyes) were tested for the presence of Betanodavirus. Pooled samples were homogenized with Earle’s balanced salt solution (Hyclone Laboratories Inc., Logan, Utah, USA) supplemented with antibiotics (amphotericin B 200 µg ml^−1^, gentamycin 500 µg ml^−1^, penicillin 1000 units ml^−1^ and streptomycin 1000 units ml^−1^) using a Tissue Master 125 (OMNI International, Kennesaw, Georgia, USA). After centrifugation of the homogenates at 2000*g* for 20 min at 4 °C, the supernatants were transferred to new tubes and incubated for 6 h at room temperature. Next, supernatants were inoculated (diluted at 10^−1^ and 10^−2^) in duplicate onto 48-well plates of semiconfluent E-11 monolayers. Infected cells were incubated at 25 °C and examined daily for the presence of CPE. After 10 days, positive and negative samples were subcultivated (by inoculating 100 µl of the scraped cell suspension onto new cultures) for 15 days.

### 2.7. Viral quantification by reverse transcription real-time PCR

Betanodavirus RNA was extracted using Nucleospin^®^ RNA (Macherey-Nagel, Duren, Germany) following the manufacturer’s instructions and reverse transcribed using a *RevertAid First Strand cDNA Synthesis Kit* (Thermoscientific Inc., Vilnius, Lithuania) in a thermocycler *MyCycler™* (Bio-Rad, Hercules, California, USA). Briefly, cDNA was synthesized in the presence of random primers (200 nM) at 42 °C for 1 h, followed by enzyme inactivation at 70 °C for 10 min. The viral load in each sample was quantified by qPCR reactions carried out in a final volume of 20 µl, containing 2 µl of cDNA, 10 µl of iQ™ SYBR^®^ Green Supermix (Bio-Rad) and 200 nM of SnodR1 F/R primers (Olveira et al. 2013). Amplification was performed in a CFX96™ Real-Time PCR Detection System (Biorad). After an initial denaturation and enzyme activation at 95 °C for 3 min, the mixture was subjected to 40 cycles of amplification (denaturation for 15 s at 95 °C, annealing-extension for 20 s at 59 °C). Quantifications were accomplished using a standard curve generated with 20-fold dilutions of SpSsIAusc160.03 RNA1 prepared from 2.91 × 10^−7^ RNA copies ml^−1^ up to 2.27 × 10^−2^ RNA copies ml^−1^ in nuclease-free water.

### 2.8. Statistical analysis

Statistical analyses were carried out using GraphPad Prism 8.1 (San Diego, California, USA). Viral quantification (TCID_50_ and RNA1 copies) data were analysed by two-way ANOVA and Tukey correction, and mortality analyses were undertaken with survival tables using the Kaplan–Meyer test. To determine significative differences between groups the Log-rank (Mantel–Cox) test was performed. *p* < 0.05 was considered statistically significant.

## 3. Results

### 3.1. Immersion and oral challenges in sole larvae

**Figure 1.**
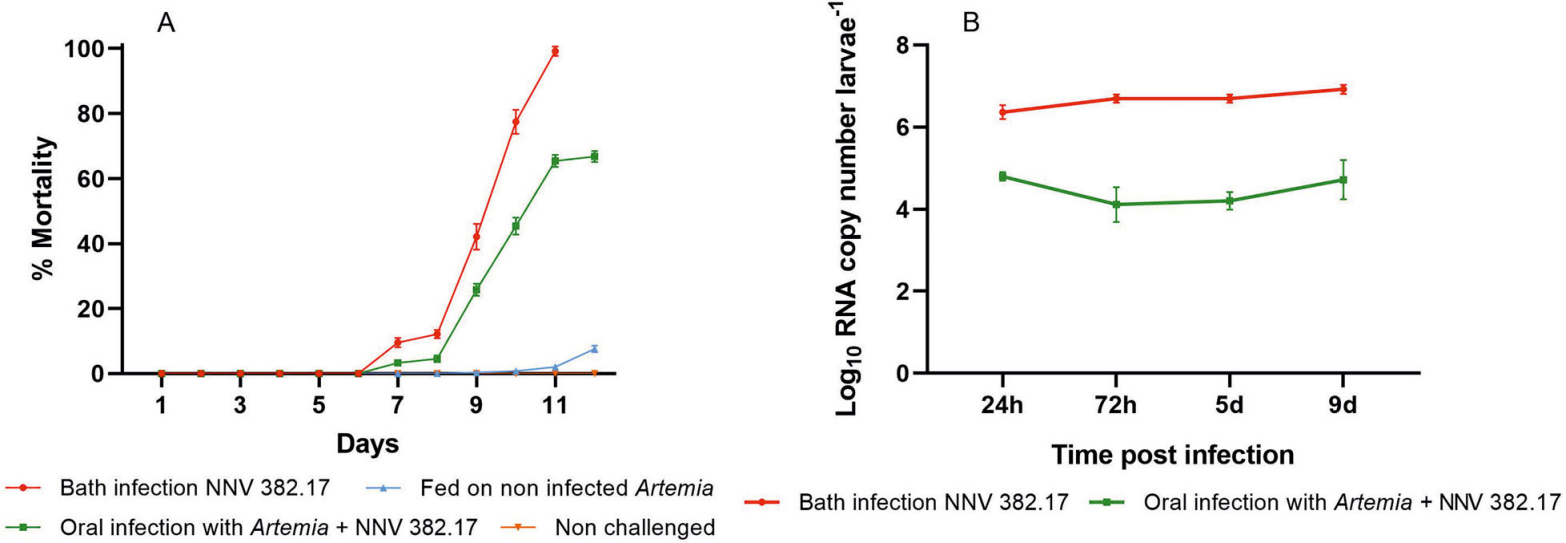
Mortality observed in the Senegalese sole larvae infected with NNV 382.17 (A) red: immersion challenge; green: oral challenge with *Artemia* carrying NNV; blue: sole fed with non-infected *Artemia*; orange: sole larvae control, and viral load quantified in sole (B) waterborne infected (red) and fed with *Artemia* carrying NNV (green). Data are presented as mean ± SD (*n* = 3).

First mortalities (4% and 12% in oral and bath infection, respectively) were recorded at day 7 post infection (pi) in both challenges, reaching 27% in sole larvae fed with *Artemia* carrying NNV and 45% in the bath-infected individuals on day 9 pi. Mortality peaked at 11 dpi, with a cumulative mortality of 66% and 100% in the oral and bath infection, respectively (Figure 1(A)). In addition, an abnormal swimming pattern was first observed in bath challenged larvae at 9 dpi, whereas in the oral infection it was observed one day later. Neither signs of disease nor deaths were observed in the bath control. Although a slight mortality (9%) was detected in the fish fed with non-infected *Artemia*, all individuals were asymptomatic and NNV was not detected in these larvae. Mortality curves in the challenged larvae were significantly different from the control group according to the Log-rank (Mantel–Cox) test (*p* value 0.0003).

Regarding the viral load at 24 hpi, orally infected individuals contained 9.34 × 10^4^ copies of viral RNA larvae^−1^, whereas the bath infection yielded 2.05 × 10^6^ RNA copies larvae^−1^ (Figure 1(B)), representing a difference of 1.34 logs. Despite mortality rates increasing over time, the number of NNV RNA copies detected in sampled larvae did not show significant variations (8.97 × 10^4^ and 9.33 × 10^6^ at 9 dpi for oral and bath challenge, respectively) and the difference between both routes of infection remained constant. The viral load quantified in the dead larvae from both oral and bath challenge (3.19 × 10^5^ and 1.67 × 10^7^ at 7 dpi and 4.82 × 10 ^5^ and 4.90 × 10^7^ at 11 dpi) was similar to that observed in the sampled fish. The number of RNA copies in the survivors of the oral challenge did not show differences with that obtained from larvae sampled at 9 dpi.

### 3.2. IFA detection of NNV in A. salina nauplii and rotifers

**Figure 2.**
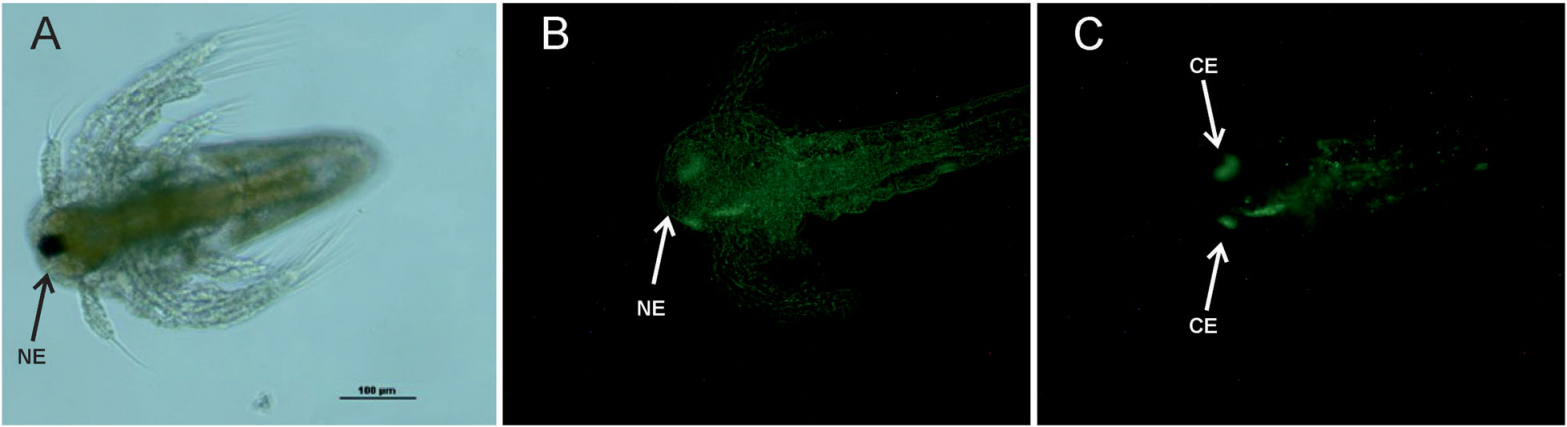
Immunofluorescence staining of *Artemia* nauplii infected with NNV 382.17 for 24 h and cultivated in sterile sea water for 48 h; bright light (A), FITC fluorescence and bright light (B), FITC fluorescence (C). Scale bar indicates 100 µm. NE: nauplius eye, CE: preliminary cells forming the compound eyes.

In *Artemia*, specific fluorescence was mainly localised in the head region. At this larval stage, the nauplius eye (NE) is already differentiated (Figure 2(A)) (Wildt and Harzsch 2002) but no virus was observed in it (Figure 2(B)). Fluorescence was concentrated behind the NE (Figure 2(C)) where cells forming the compound eye (CE) are located (Criel and Macrae 2002; Wildt and Harzsch 2002). To a lesser extent, it was also detected all along the animal, probably due to unspecific antibody binding to the chitinous exoskeleton.

**Figure 3.**
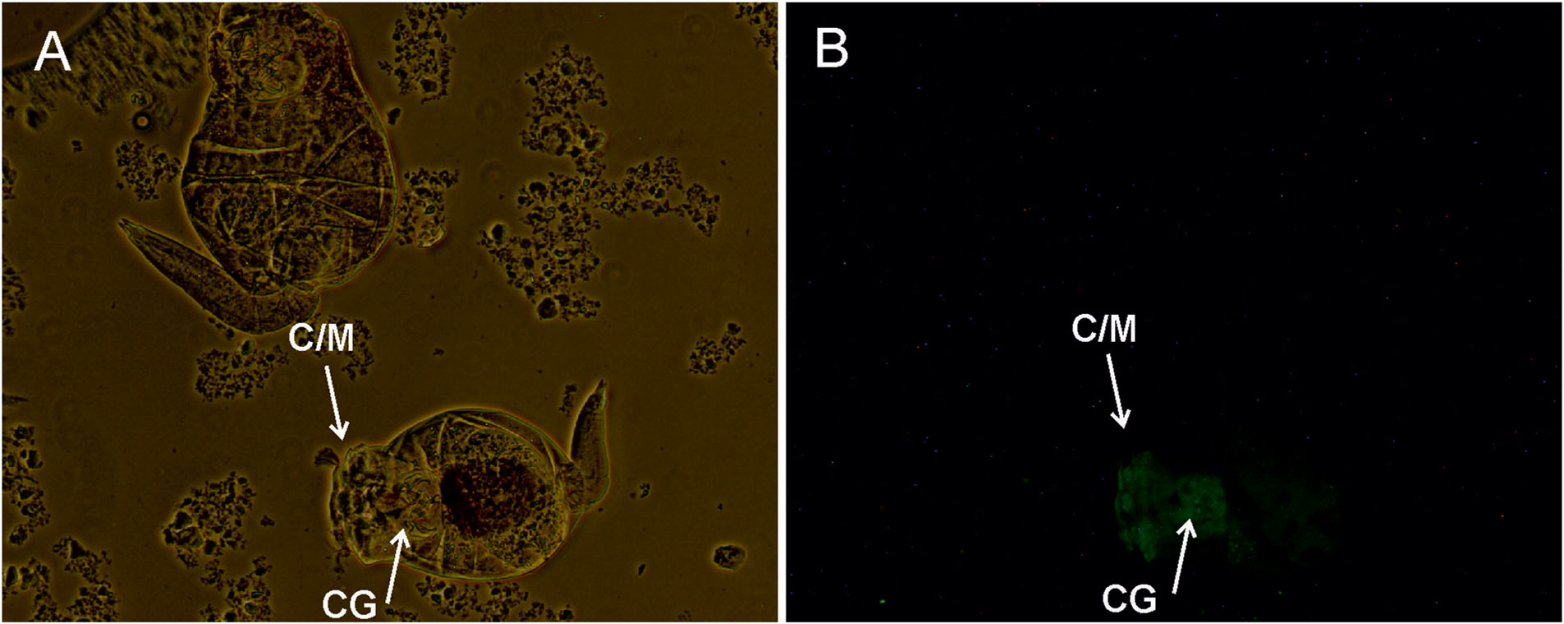
Immunofluorescence staining of rotifers infected with NNV 382.17 for 24 h and cultivated in sterile sea water for 48 h, bright light image of two female rotifers (A) and fluorescence observed in one of them (B). C/M: corona and masticatory organ; CG: cerebral ganglia.

In rotifers, fluorescence was concentrated in the anterior region (Figure 3(B)) where the corona (C), the masticatory organ (M) and cerebral ganglia (CG) (Figure 3(A)) are located (Boell and Bucher 2008) and, to a lesser extent, in the perivisceral fluid along the whole body.

### 3.3. NNV quantification in rotifers and A. salina nauplii

**Figure 4.**
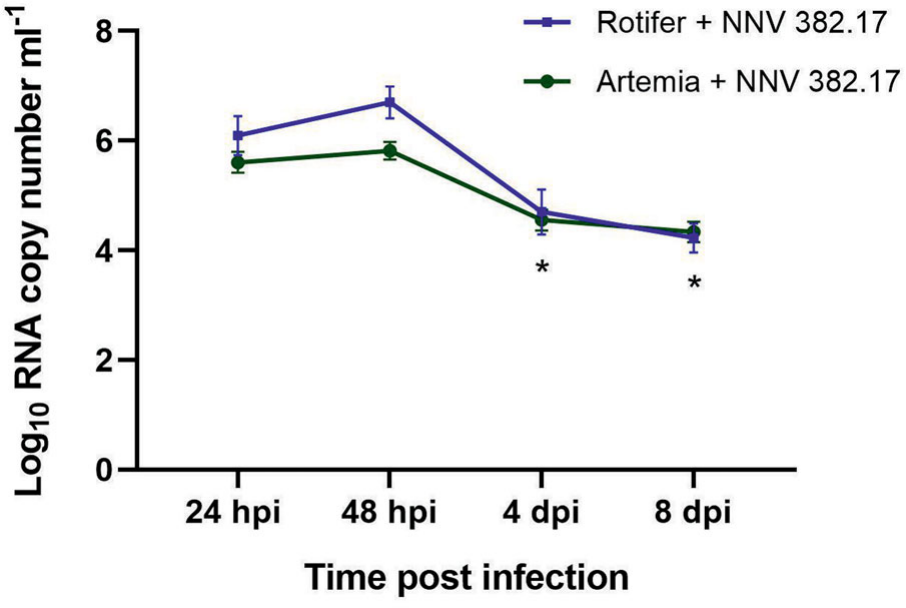
Viral load quantified into *Artemia* nauplii (green) and rotifer (blue) waterborne infected with NNV 382.17 with no food supply. Data presented as mean ± SD (*n* = 3). Asterisks show significant differences compared to the 24 hpi samples (*p value < 0.05).

NNV RNA was detected in the infected zooplankton until the end of the experiment (8 dpi). At 24 hpi, the mean RNA copy number was 1.52 × 10^6^ ml^−1^ in rotifers and 2.88 × 10^5^ ml^−1^ in *Artemia* and no significant differences were observed when compared with the inoculum (*p* value > 0.05). At 48 hpi, a non-significant increase (5.71 × 10^6^ in rotifers and 7.46 × 10^5^ in *Artemia* samples, *p* value > 0.05) was detected (Figure 4). Although at 4 dpi, a significant reduction in the number of NNV RNA copies was observed (1.32 × 10 ^5^ in rotifers and 6.78 × 10^4^ in *Artemia*; *p* value < 0.05), at 8 dpi, the viral load inside the invertebrates was still moderate (1.92 × 10^4^ in rotifers and 1.97 × 10^4^ in *Artemia*). The virus was recovered in cell culture at 24 and 48 hpi from rotifers and up to 4 dpi from *Artemia*.

**Figure 5.**
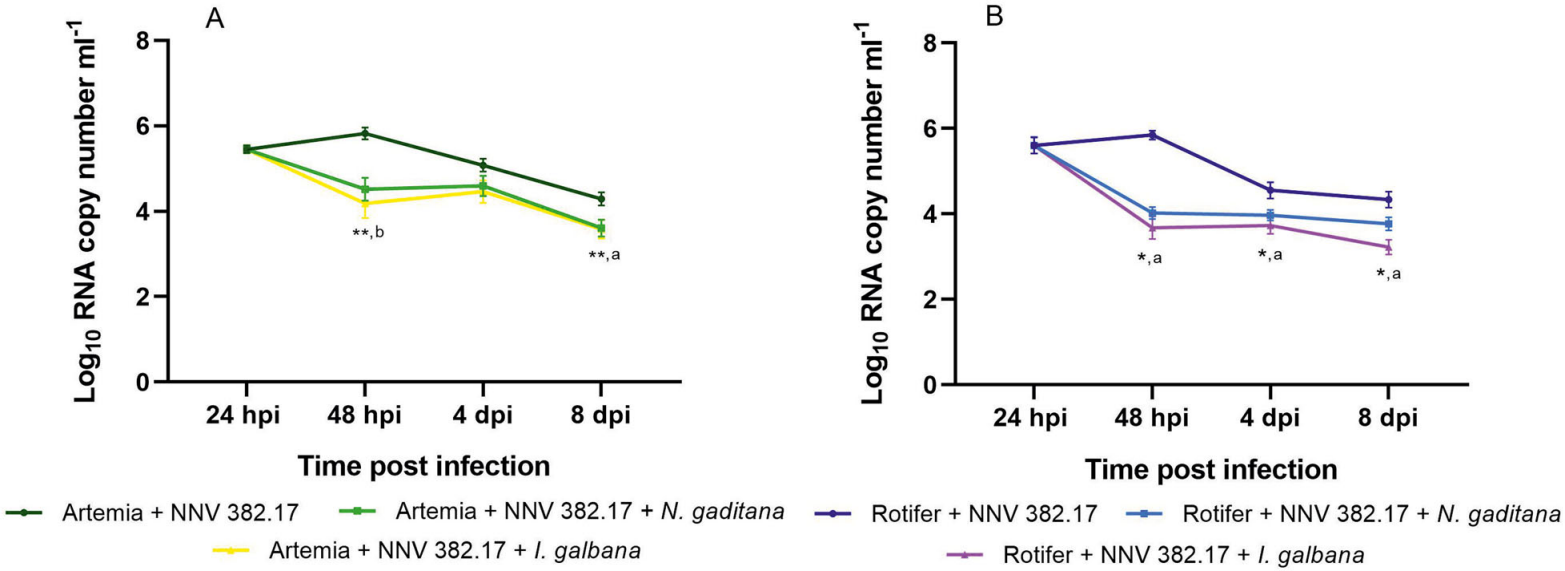
Viral load quantified in *Artemia* (A) waterborne infected for 24 h when no food was added (dark green) and fed on *N. gaditana* (light green) and *I. galban*a (yellow), and in rotifers (B) with no food (dark blue) and fed on *N. gaditana* (light blue) and *I. galban*a (purple)_._ Data presented are the mean values ± SD of three replicates. Asterisks indicate significant differences compared with the 24 hpi samples (*p value < 0.05, **p value < 0.001). Letters show significant differences with the unfed group at given time points (a, *p* value < 0.05, b, *p* value < 0.001).

No alterations in the swimming behaviour were observed in any of the invertebrates, suggesting that they were not suffering from neurological damage. However, from day 4 pi onwards, some mortality was observed in both invertebrates, which was attributed to the effects of starvation. Therefore, three new infection assays were performed in triplicate using invertebrates both unfed and fed either with *N. gaditana* or *I. galbana*, in order to study if the reduction in viral load was related to zooplankton mortality. As occurred in the previous experiment, at 24 hpi the viral load (3.95 × 10^5^) was similar to the inoculum in the unfed *Artemia* nauplii, and a non-significant increase (0.41 logs; *p* value > 0.05) was observed at 48 hpi. However, when fed on *N. gaditana* and *I. galbana*, there was a decrease of 1.34 and 1.27 logs, respectively (*p* value 0.0007) (Figure 5(A)). Finally, at 8 dpi, a significant decrease in the number of NNV RNA copies compared to the 24 hpi samples was observed (*p* value 0.0001) and the difference between the fed and unfed groups was 0.74 logs (*p* value < 0.05). Similar results were obtained in rotifer cultures, which carried an initial viral load of 1.46 × 10^5^ copies ml^−1^ (Figure 5(B)). In the unfed groups, the RNA copy number showed a significant reduction at 4 dpi, which was maintained at 8 dpi (1.05 and 1.27 logs, respectively). However, the number of NNV RNA copies decreased significantly 24 h after being fed on *N. gaditana* (48 hpi) (1.58 logs; *p* value 0.0072), and an even higher reduction was observed after feeding with *I. galbana* (1.93 logs; *p* value 0.0441). The differences in the viral load between unfed and fed individuals were reduced at 4 dpi but increased significantly on day 8 pi in individuals fed with *I. galbana* with respect to the unfed ones (1.11 logs, *p* value 0.0036). The isolation of infective particles in cell culture was achieved from *Artemia* samples up to 48 hpi (from both fed and unfed individuals). However, when rotifer samples were analysed, viral recovery was only accomplished from unfed individuals at 24 hpi. No VER signs were observed in any of the challenged invertebrates.

**Figure 6.**
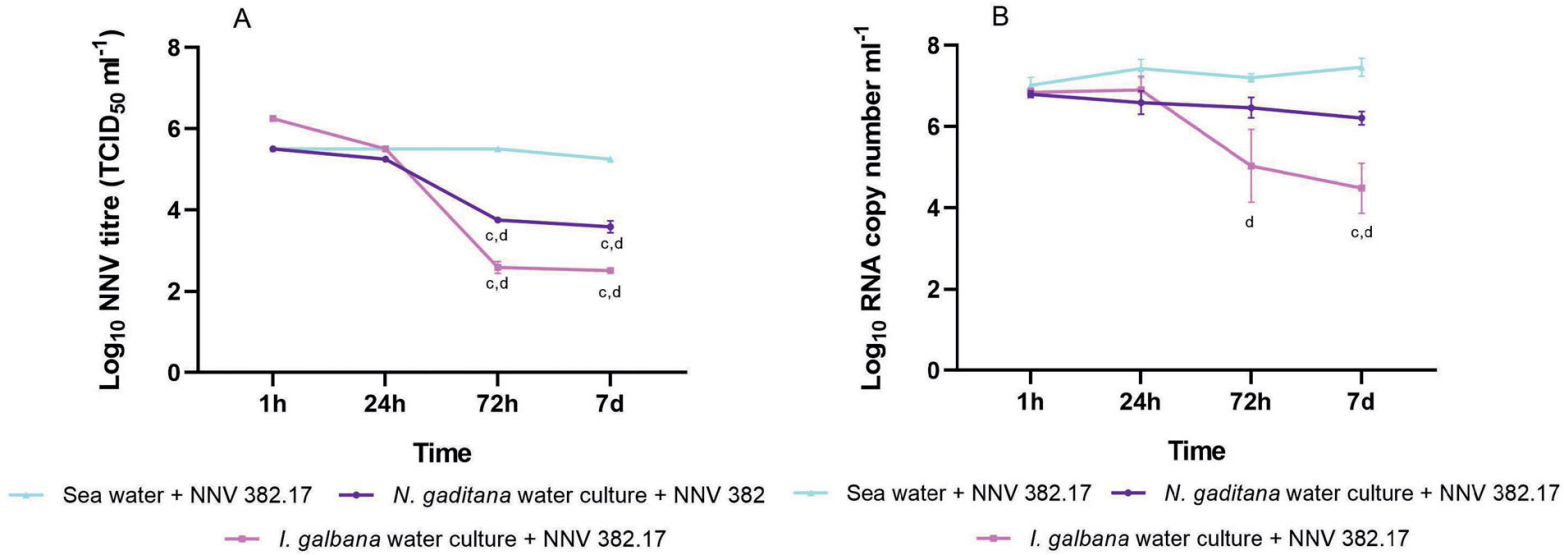
Viral quantification expressed as TCID_50_ ml^−1^ (A) and RNA copies ml^−1^ (B) after incubating the 382.17 strain in microalgae water culture (purple: *N. gaditana*; pink: *I. galbana*) and in sea water (light blue). Data are presented as mean ± SD (*n* = 3). Letters indicate significant differences with the 24 h samples (c, *p* value < 0.001) and with sea water (d, *p* value < 0.001).

Given that feeding the invertebrates with microalgae did not reduce the decrease in the viral load but rather increased it, we decided to investigate the effect of the microalgae culture on virus viability. To this end, 382.17 was incubated in *N. gaditana* and *I. galbana* water culture and the viral survival was compared with that in sea water. As expected, NNV incubation in sea water for 7 days did not result in a reduction in viral titre, but it was clearly reduced when incubated for 72 h in *N. gaditana* water culture (almost 2 logs, *p* value 0.0009) and even more so in the water obtained from *I. galbana* (more than 3.5 logs, *p* 0.0001) (Figure 6(A)). A similar effect was observed in the number of viral RNA copies with a reduction of 2.27 logs (*p* value 0.0002) 7 days after incubation with *I. galbana* water culture (Figure 6(B)), whereas *N. gaditana* caused a non-significant reduction (0.59 logs; *p* value 0.1239).

## 4. Discussion

Horizontal NNV transmission from fish to fish or through the water body, has been fairly demonstrated in a number of fish species (Bandín and Souto 2020). In addition, putative viral transmission to marine larval stages through live food (rotifers and brine shrimp) has been suggested (Skliris and Richards 1998). In the present report, successful horizontal NNV transmission and VER development has been achieved in Senegalese sole larvae fed with infected brine shrimp, *A. salina*.

The sole larvae (30 dph) were fed once with infected *Artemia* nauplii and NNV transmission was demonstrated after viral recovery from larval brain tissue. In addition, VER development was evidenced by larval abnormal swimming behaviour and a 66% recorded mortality. As expected, mortality and viral load were lower than in the bath challenge used as a positive control, probably due to the different ways the virus enters the host. Whereas NNV present in water can reportedly use gills, skin and the oral cavity as portals of entry to sole juveniles (Souto et al. 2018), through live food NNV can only get into the larvae using the digestive tract. Therefore, VER development in the sole larvae was probably triggered once NNV was released from the *Artemia* during the digestion process and finally reached larvae brain tissue either through the nervous or the circulatory systems (Bandín and Souto 2020). This experiment suggests that cell receptors responsible for viral attachment to brain and retina in adults are already present in 30 dph sole larvae as reported for other fish species, such as grouper (*Epinephelus* sp), striped jack (*Pseudocaranx dentex*), Atlantic halibut (*Hippoglossus hippoglossus*) and European sea bass (Mori et al. 1991; Nguyen et al. 1996; Grotmol et al. 1999; Breuil et al. 2002). Unfortunately, oral infection through rotifers, which are used as a first larval feed, was not successful because the appropriate larval stage (2–3 dph) did not survive in our aquarium facilities. However, NNV was detected in the rotifers up to 8 dpi, suggesting that these marine invertebrates could also act as viral vectors. Previous studies have reported the horizontal transmission of different pathogens through both invertebrates used as live food either in finfish or shellfish cultures. Rotifers have been reported to contain potential fish pathogenic bacteria (Turgay et al. 2020) and to transmit viral pathogens to shellfish, such as the White Spot Syndrome Virus (WSSV) to crayfish (*Procambarus clarkii*) and the black tiger shrimp, *Penaeus monodon* (Zhang et al. 2006; Yan et al. 2007; Corre et al. 2012), and the Covert Mortality Nodavirus (CMNV) to the Pacific white shrimp *Litopenaeus vannamei* (Liu et al. 2018). *Artemia* has also been demonstrated to transmit CMNV, *Baculovirus penaei* and Infectious Myonecrosis virus (IMNV) to the Pacific white shrimp (Overstreet et al. 1988, Silva et al. 2015; Liu et al. 2018), *Macrobachium rosembergii* nodavirus (MrNV) to giant freshwater prawn (Sudhakaran et al. 2006), and Lymphocystis disease virus (LCDV) to gilthead seabream larvae (Valverde et al. 2019).

Both rotifer and *Artemia* display a filter-feeding behaviour characterized by filtering non-selected particles smaller than 50 µm (Kiørboe 2011) which could lead to the accumulation of NNV particles and explain their role as NNV vectors. Our results demonstrate that NNV can be internalised and accumulated in both invertebrates after the immersion challenge. In rotifers, fluorescence was observed in the corona and the masticatory organ, where different neurons are connected (Kotikova et al. 2005), and in the cerebral ganglia. In *Artemia*, IFA results showed NNV attachment to the preliminary cells forming the adult compound eyes, located between the nauplius eye and the first antennae (Criel and Macrae 2002). The decrease in the viral load through the experimental course indicates that no viral replication is being produced, although infective viral particles were recovered in cell culture 48 hpi from rotifers and up to 4 dpi from *Artemia*. Despite this decrease, however, viral clearance was not achieved in any of the invertebrates, supporting their role as NNV-biological vectors. NNV accumulation has already being reported in bivalve molluscs (Volpe et al. 2017, 2018; Kim et al. 2018; Bitchava et al. 2019) and viral detection in other molluscs and crustaceans (Gomez et al. 2006, 2008; Fichi et al. 2015) suggests that marine invertebrates are also a potential source of NNV in the wild.

None of the challenged invertebrates showed typical VER symptomatology, like looping or resting belly up, as previously demonstrated by Skliris and Richards (Skliris and Richards 1998). The lack of symptoms in brine shrimp challenged with IMNV (Silva et al. 2015), WSSV (Li et al. 2003) and MrNV (Sudhakaran et al. 2006) has already been reported. Furthermore, the replication of LCDV at different growth stages of the invertebrate did not provoke disease symptoms (Valverde et al. 2019). However, in our challenge, mortality was recorded in both rotifers and *Artemia* 4 dpi onwards; this prompted us to perform additional experiments in order to assess whether mortality was due to viral accumulation or to the starvation effects on the invertebrates’ body functions, as they were not fed during the challenge. In these new assays, the invertebrates were fed with microalgae after viral infection. Phytoplankton is broadly utilized in aquaculture as nutritional live food for zooplankton, providing them with docosahexaenoic acid (DHA), eicosapentaenoic acid (EPA) and free amino acids (FAA) among others (Eryalçın 2018, 2019; Ferreira et al. 2018). *N. gaditana* and *I. galbana* are two of the most commonly used species (Hemaiswarya et al. 2011) and were therefore used as the food supply for our invertebrate cultures. Interestingly, the viral load in the fed groups was even lower than that of the unfed ones, regardless of the microalgae used, suggesting that feeding the invertebrates with both microalgae species has a negative impact on NNV viability. It has been demonstrated that several microalgae produce and release compounds with strong antibacterial and antiviral activity (Falaise et al. 2016), therefore, in order to check the secretion of any antiviral substances by the microalgae species used in this study, NNV was incubated for 7 days in the supernatant obtained after centrifuging *N. gaditana* and *I. galbana* cultures at the stationary phase and then viral survival was compared with that in seawater. NNV incubation in seawater did not result in any viral viability alteration as previously reported (Frerichs et al. 2000). However, both microalgae supernatants caused a significant reduction in the TCID_50_ values (2 and 3.5 logs in the case of *N. gaditana* and *I. galbana*, respectively), but genomic values decreased only after incubation with *I. galbana*. These results indicate that although both microalgae can affect viral infectivity, different mechanisms must be involved in their inhibitory properties. As previously reported, microalgae metabolites can block viral adsorption/penetration (Hernández-Corona et al. 2002), but other steps of viral replication can also be affected (Huheihel et al. 2002). Studies are in progress to characterize the anti-NNV compound secreted by both microalgae and the mechanism involved in their antiviral activity.

On the other hand, the NNV reassortant strain used in this study was isolated from diseased gilthead seabream and turned out to be highly pathogenic to waterborne infected sole larvae (100% mortality), demonstrating that horizontal transmission between these two species is possible, as recently proved between gilthead sea bream and European sea bass, although causing low mortality in the second species (Volpe et al. 2020). So far, the RGNNV/SJNNV reassortant strains have only been isolated in Southern Europe, from both farmed and wild fish species (Toffolo et al. 2007; Olveira et al. 2009; Toffan et al. 2017; Bitchava et al. 2019; Volpe et al. 2020). Our results suggest that interspecies transmission could be involved in the spread of reassortant strains in the wild.

## 5. Conclusion

We have demonstrated that live food can be a vector of horizontal NNV spreading to *Solea senegalensis* larvae and that inter-species transmission of reassortant strains is possible. Although VER developed only in sole larvae orally challenged with infected *Artemia*, both rotifers and *Artemia* can be considered NNV vectors, therefore becoming a potential threat to aquaculture production. In addition, we have shown that feeding these invertebrates with both microalgae, but specially with *I. galbana,* not only provides them with the suitable biochemical composition to become a proper feed for optimal fish larvae growth, survival and normal development, but also gives them some protection against viral infections. All of these results can contribute to the development of prophylactic measures to be used in aquaculture hatcheries.
